# Genomewide transcriptional response of *Escherichia coli* O157:H7 to norepinephrine

**DOI:** 10.1186/s12864-021-08167-z

**Published:** 2022-02-08

**Authors:** Vijay K. Sharma, Suryatej Akavaram, Darrell O. Bayles

**Affiliations:** 1 Food Safety and Enteric Pathogens Research Unit, National Animal Disease Center, ARS-USDA, Ames, IA 50010 USA; 2 Current address: 4302 TX-332, Freeport, TX 77541 USA; 3 Infectious Bacterial Diseases Research Unit, National Animal Disease Center, ARS-USDA, Ames, IA 50010 USA

**Keywords:** O157, Norepinephrine, Acid resistance, Two-component signaling pathways, Adherence

## Abstract

**Background:**

Chemical signaling between a mammalian host and intestinal microbes is health and maintenance of ‘healthy’ intestinal microbiota. *Escherichia coli* O157:H7 can hijack host- and microbiota-produced chemical signals for survival in a harsh and nutritionally competitive gastrointestinal environment and for intestinal colonization. Norepinephrine (NE) produced by sympathetic neurons of the enteric nervous system has been shown in vitro to induce expression of genes controlling *E. coli* O157:H7 swimming motility, acid resistance, and adherence to epithelial cells. A previous study used a microarray approach to identify differentially expressed genes in *E. coli* O157:H7 strain EDL933 in response to NE. To elucidate a comprehensive transcriptional response to NE, we performed RNA-Seq on rRNA-depleted RNA of *E. coli* O157:H7 strain NADC 6564, an isolate of a foodborne *E. coli* O157:H7 strain 86–24. The reads generated by RNA-Seq were mapped to NADC 6564 genome using HiSat2. The mapped reads were quantified by htseq-count against the genome of strain NADC 6564. The differentially expressed genes were identified by analyzing quantified reads by DESeq2.

**Results:**

Of the 585 differentially expressed genes (≥ 2.0-fold; *p* < 0.05), many encoded pathways promoting ability of *E. coli* O157:H7 strain NADC 6564 to colonize intestines of carrier animals and to produce disease in an incidental human host through increased adherence to epithelial cells and production of Shiga toxins. In addition, NE exposure also induced the expression of genes encoding pathways conferring prolonged survival at extreme acidity, controlling influx/efflux of specific nutrients/metabolites, and modulating tolerance to various stressors. A correlation was also observed between the EvgS/EvgA signal transduction system and the ability of bacterial cells to survive exposure to high acidity for several hours. Many genes involved in nitrogen, sulfur, and amino acid uptake were upregulated while genes linked to iron (Fe^3+^) acquisition and transport were downregulated.

**Conclusion:**

The availability of physiological levels of NE in gastrointestinal tract could serve as an important cue for *E. coli* O157:H7 to engineer its virulence, stress, and metabolic pathways for colonization in reservoir animals, such as cattle, causing illness in humans, and surviving outside of a host.

**Supplementary Information:**

The online version contains supplementary material available at 10.1186/s12864-021-08167-z.

## Background

*Escherichia coli* O157:H7 (O157) infections in healthy human adults usually result in an asymptomatic and self-resolvable watery diarrhea [1]. However, in children and elderly individuals, O157 infections can lead to development of more serious symptoms such as abdominal pain, bloody diarrhea or hemorrhagic colitis, and hemolytic uremic syndrome [2, 3]. Besides producing Shiga toxins, which are a major cause of kidney failure and even death in infected humans [4, 5], O157 strains encode virulence factors that promote their ability to colonize the large intestine of incidental human hosts and carrier animals, such as cattle [6]. These adherence-promoting virulence factors are secreted through a type-3 secretion system, genes for which are encoded by a pathogenicity island, called the locus of enterocyte effacement (LEE) [6–9]. LEE is composed of five major operons and three of these five operons (*LEE1* – *LEE3*) are involved in formation of the type three system which secrets adhesin intimin, intimin receptor called translocated intimin receptor (Tir), and many other proteins involved in the formation of attaching and effacing lesions on intestinal mucosa [10, 11]. Although LEE expression is positively regulated by Ler [11], several positive and negative transcriptional regulators, some LEE- and some non-LEE-encoded, and a variety of bacterial, host, and environmental signals control Ler expression to ensure optimal LEE expression occurs in the appropriate intestinal compartment [12, 13].

Since the preferred site for O157 colonization in ruminants, such as cattle, is the terminal colon, specifically the rectoanal junction (RAJ) [7, 8], O157 first traverses the highly acidic environment of the abomasum before reaching RAJ. Several studies have shown that the ability to sense a variety of chemical signals and metabolites produced by the host and intestinal microbiota play an important role in the survival of O157 at the extreme acidic pH of the stomach and subsequent O157 colonization at the RAJ. For example, in cattle, which are the primary reservoir for O157 and source of O157 infections in humans, bacterial members of the rumen microbiota produce acyl-homoserine lactones that are perceived as a quorum-sensing (QS) signal by a LuxR homolog SdiA to induce expression of glutamate-dependent acid resistance pathway 2 (ARP2) [14, 15]. ARP2 ensures survival of O157 at a very low pH (pH 2) and thus accounts for the low infectious dose of O157 in human infections [14, 16–18]. The expression of ARP2, which uses glutamate decarboxylase A (GadA) and GadB, and an antiporter (GadC) to confer acid resistance, is regulated by GadE [19]. GadE expression is controlled by transcriptional regulators GadX, GadW, and a two-component signal transduction system EvgS/EvgA [20–22]. EvgS/EvgA responds to low pH and alkali metals to regulate acid resistance and multidrug resistance efflux pumps in *E. coli* [23]. The response regulator EvgA has been shown to induce *gadE* transcription either through the induction of YdeO, which interacts with the *gadE* promoter, or through direct interaction of EvgA at an undefined site in the *gadE* promoter [22, 24].

While the sensing of acyl-homoserine lactones enhances expression of ARP2 by GadE, LEE expression is repressed by GadE since LEE functions are not needed in the rumen. To colonize the RAJ, O157 uses different QS pathways to sense signals, such as autoinducer-3 (AI-3) produced by many bacterial species of intestinal microbiota [25–28], and host-produced stress hormones norepinephrine/epinephrine [29–31]. About half of norepinephrine (NE) is synthesized and utilized locally within the enteric nervous system by adrenergic neurons in the basal-lateral layer of the gut, epinephrine (E), on the other hand, is mostly synthesized in the adrenal medulla and reaches the small intestine via blood [30, 32, 33]. According to many studies, NE not only enhances growth, iron acquisition, motility and Shiga toxin expression, but also induces acid resistance and promotes adherence of O157 to epithelial cells [34–40]. The mechanism for NE-mediated growth and virulence enhancement of O157 is linked to the release of iron (Fe^3+^) from transferrin and lactoferrin, which are important innate immune defense proteins in mammalian hosts [35, 41, 42]. NE and NE-Fe^3+^ complexes reach the periplasm through the outer membrane embedded ferric iron transport system, and outer membrane proteins OmpA, and OmpC [35, 42]. Once in the periplasm, NE is sensed by the inner membrane-embedded quorum-sensing signal transduction systems (QseBC and QseEF) that through a phospho-relay cycle leads to the activation of a cascade of response regulators, which induce expression of motility, LEE, and Shiga toxins [30]. In addition to impacting gene expression directly, NE is also metabolized by commensal *E. coli* to 3,4-dihydroxymandelic acid (DHMA), which serves as a bacterial chemoattractant, induces the expression of virulence genes, and enhances attachment of O157 to intestinal epithelial cells in a QseC-dependent manner [27].

A recent study that used a probe set of 610 genes in a microarray-based transcriptional profiling of O157 strain EDL933 demonstrated that NE enhanced expression of genes involved in tissue adherence, Shiga toxin production, motility, ARP2, and reduced expression of genes for iron acquisition [34]. In the current study, we describe the use of RNA-Seq to determine the differential gene expression profile of O157 strain NADC 6564 [43] when grown in the presence of NE. Like the previous microarray-based gene expression profiling [34], we found that NE-mediated signaling resulted in the differential expression of genes encoding pathways for survival at a very low pH and for intestinal colonization. We also showed that NE-mediated induction of the EvgS/EvgA signaling system is directly linked to the expression of acid resistance phenotype. In addition, we demonstrated that exposure of strain NADC 6564 to NE resulted in the repression of enterobactin siderophore biosynthesis used for iron (Fe^3+^) acquisition but enhanced the expression of ferrous uptake pathway that is more active under low pH and anaerobic conditions. The differential regulation of numerous other pathways, such as those controlling transport of amino acids and peptides, salvage of pyrimidines, storage and utilization of carbohydrate substrates, nitrogen and sulfur metabolism, and sensing of various stressors indicated that strain NADC 6564 uses NE to alter its cellular physiology and cell membrane functions that in all likelihood are advantageous for O157 survival, growth, and colonization of specific sites in the large intestinal compartment of its carrier animal and the incidental human host.

## Results

### Large numbers of genes were upregulated in response to norepinephrine

**Fig. 1:**
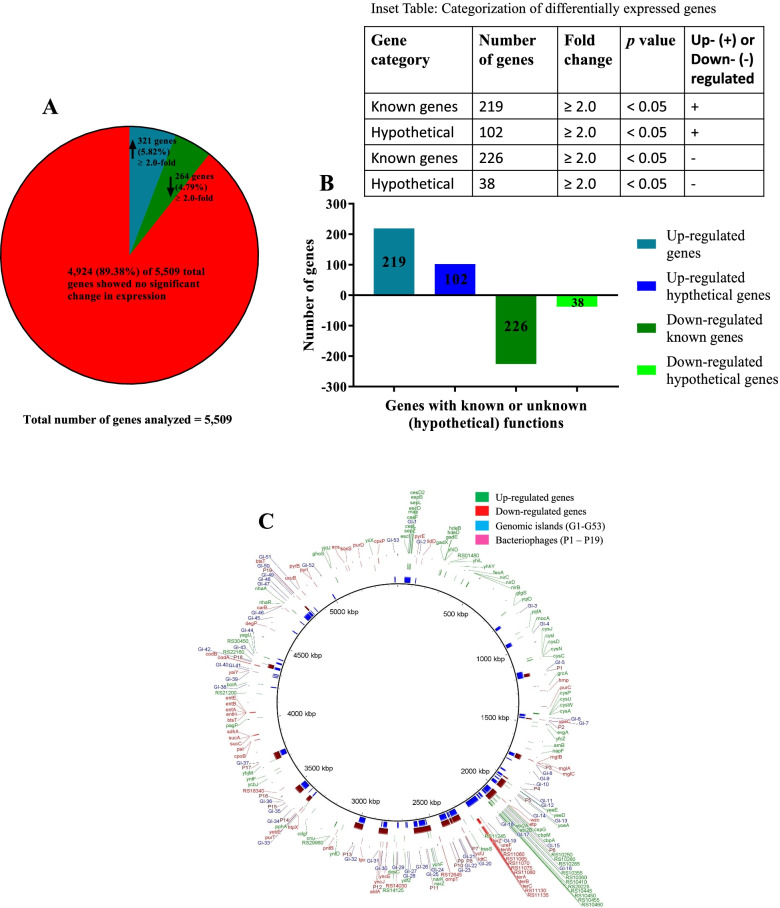
Graphical representation of differentially expressed gene by *E. coli* O157:H7 strain NADC 6564 in response to norepinephrine (**A**) A pie chart showing proportion of significantly upregulated (dark blue slice), downregulated (light blue slice), and unaffected genes (red slice) in total of 5509 chromosomal genes analyzed by RNA-Seq, (**B**) A bar graph showing number of upregulated genes with known function (dark blue bar), upregulated genes assigned hypothetical function (light blue bar), downregulated genes (dark green bar), and downregulated genes assigned hypothetical function (light green bar). Inset Table in Fig. 1 shows up- and downregulated genes of known and hypothetical functions and their fold change in expression, and (**C**) BRIG plot showing distribution of upregulated genes (green), downregulated genes (red), Genomic islands 1–53 (blue) and bacteriophages P1 – P19 (purple) on the chromosome of strain NADC 6564. Chromosomal size (5,466,770 bp) of strain NADC 6564 is listed in the center of the inner circle, which is marked on the inside using a 500 kbp (kilo base pairs) scale

The results described below are based on a genome-scale transcriptomic analysis of *E. coli* O157H:H7 (O157) strain NADC 6564 [43] in response to NE that overcomes the limitations of a previously reported study involving only a 610 gene array to determine the differential response of *E. coli* O157:H7 strain EDL933 to norepinephrine (NE). Although some of the major findings of the current study were similar to the 610 gene array-based study, we identified many other differentially expressed genes regulating a variety of pathways in O157 in response to NE. We used an FDR-adjusted *p*-value of 0.05 and ≥ 2.0-fold change in gene expression as a threshold for considering a gene being differentially expressed (DE) in NE-treated relative to untreated bacterial cultures. Many of the genes that we identified as DE at ≥2.0-fold, *p* ≤ 0.05 in response to NE (this report) were also identified as DE in a previously reported microarray-based study that used a threshold of 1.5-fold for a gene to be considered DE [34]. RNA-Seq analysis of the genome-scale transcriptome of the norepinephrine (NE)-treated and untreated cultures of strain NADC 6564 allowed us to determine the proportion of DE genes. Overall, 5509 genes (Fig. 1A, Table S1), representing 98.76% of 5578 total genes predicted in the chromosomal sequence of NADC 6564 [43], generated reads mapping to the reference genome. Using the ≥2.0-fold threshold, 585 genes, representing 10.6% of 5509 genes, were DE (*p* > 0.05) in response to NE (Fig. 1A). Of these 585 DE genes, 321 genes (about 5.82%) were upregulated and 264 genes (about 4.79%) were downregulated in NE-treated cultures (*p* < 0.05) (Fig. 1A and Inset Table in Fig. 1; Table S2 and Table S3). About 31.8% (102 of 321 genes) of the upregulated genes and about 14.4% (38 of 264 genes) of the downregulated genes were predicted to encode hypothetical proteins with unknown functions (Fig. 1B and Inset Table Fig. 1). The DE genes were not localized to any specific region of the chromosome, rather both up- and downregulated genes were distributed randomly throughout the whole chromosome of strain NADC 6564 (Fig. 1C). However, the region of the chromosome (located between 2000 kbp – 2300 kbp) containing a bacteriophage labeled P6 and a genomic island G19 contained a cluster of several upregulated and downregulated genes, respectively, although majority of these genes encoded hypothetical proteins of unknown functions (Fig. 1C). Therefore, the biological significance of the differential expression of genes in this cluster is not clear and will depend on the functional characterization of these genes/gene products and identification of regulatory networks controlling these genes.

### Norepinephrine-treated cultures showed enhanced expression of virulence genes

Among the virulence genes, majority of LEE-encoded genes and the *stx*2 gene (encodes for Stx2 subunits A and B) were significantly upregulated (≥ 2.0-fold, *p* < 0.05) in NE-treated cultures (Table 1; Table S2). Since LEE expression is activated by LEE-encoded *ler*, and *ler* expression in turn is modulated by a network of transcriptional factors, we analyzed the RNA-Seq data to determine if NE exposure resulted in enhanced expression of *ler* and differential expression of specific LEE- and non-LEE-encoded transcriptional factors. This was done to gain insight into the mechanism of regulation of LEE by these transcriptional factors. We did not detect any change in the expression of *ler*, which encodes the transcriptional factor Ler for activating LEE expression (Table S2), However, two of the four copies of the *perC* gene, which encodes transcriptional factor PerC, were upregulated by ≥2-fold (*p* < 0.05) in NE-treated cultures (Table 1; Table S2). The four copies of *perC* are located at different chromosomal locations.

**Table 1:** Norepinephrine enhanced expression of genes encoding virulence pathways

Pathway/Gene group^**a**^	Locus tag	Gene^**b**^	Gene function	Fold change^**c**^	***p*** adjusted
**Virulence/LEE**					
	BHW77_00345	*sepD*	lytic transglycosylase	+ 2.04	2.06E-02
	BHW77_00350	*escJ*	Secreted inner membrane ring protein	+ 2.25	1.29E-02
	BHW77_00355	*escI*	Type III secretion system (T3SS) inner rod protein	+ 3.04	5.54E-06
	BHW77_00360	*sepZ*	T3SS protein	+ 2.80	1.58E-06
	BHW77_00365	*cesL*	T3SS regulator	+ 3.12	2.43E-06
	BHW77_00370	*escV*	T3SS export apparatus protein	+ 2.29	1.56E-04
	BHW77_00400	*cesF*	T3SS molecular chaperone	+ 3.12	1.31E-06
	BHW77_00405	*map*	T3SS effector protein	+ 3.88	3.24E-11
	BHW77_00430	*sepL*	T3SS gatekeeper	+ 4.49	1.52E-08
	BHW77_00435	*espA*	T3SS needle protein	+3.236	9.67E-05
	BHW77_00440	*espD*	T3SS needle protein	+2.995	6.62E-05
	BHW77_00445	*espB*	T3SS translocon pore-forming subunit	+ 3.15	6.19E-06
	BHW77_00450	*cesD*	T3SS chaperon	+ 3.06	1.12E-06
**Virulence/*stx2***					
	BHW77_10375	*stx2A*	Shiga toxin subunit A	+ 4.08	4.66E-10
	BHW77_10380	*stx2B*	Shiga toxin subunit B	+ 3.37	2.09E-09
**Virulence/LEE,** ***stx*** **transcriptional regulators**					
	BHW77_09855	*perC*	PerC family protein transcriptional regulator	+ 2.47	3.28E-04
	BHW77_18290	*perC*	PerC family protein transcriptional regulator	+ 2.57	1.23E-03
**Virulence/Fimbriae**					
	BHW77_01085	*lpfB*	long polar fimbrial chaperone LpfB	+ 2.45	1.00E-04
	BHW77_01095	*lpfD*	fimbrial family protein	+ 3.01	1.43E-06
	BHW77_20140	*ybgP*	fimbrial protein	+ 2.02	7.68E-04
	BHW77_11425	*csgB*	curli subunit protein	+ 7.85	3.20E-02

^a^Gene group/gene designations were selected from RAST Server [109]

^b^Gene name and known or predicted functions are based on the annotated sequence of *E. coli* O157:H7 EDL 933 [110]

^c^ + Symbol represents that the gene expression listed in the column was upregulated

Although LEE encoded proteins are critical for O157 adherence to epithelial cells, many other adhesins, particularly those represented by fimbriae also play an important role in adherence of O157 to mammalian tissues and to abiotic matrices to produce biofilms. RNA-Seq analyses revealed significantly higher expression of several genes belonging to Lpf1 (*lpfB* and *lpfD*), Ygp, and curli (*csgB*) fimbrial groups (Table 1 and Table S2) in response to NE. The *csgB* gene, which is located in the *csgBAC* operon [44] and encodes CsgB for nucleating CsgA subunits into curli fimbriae, showed the highest increase (+ 7.8-fold, *p* < 0.05) in its expression (Table 1), but no other genes involved in curli biogenesis were differentially expressed. A cdGMP encoding *dosC* gene was also upregulated (+ 2.86-fold, *p* < 0.05) (Table 2, Table S2) in response to NE and increased expression of *dosC* has been shown to enhance *csgB* expression and biofilm formation in *E. coli* [45].

**Table 2:** Norepinephrine enhanced expression of genes encoding various stress-related pathways

Pathway/Gene group^**a**^	Locus Tag	Gene^**b**^	Gene function	Fold change^**c**^	***p*** adjusted
**Stress/cell division/biofilms**					
	BHW77_19325	*dps*	DNA starvation/stationary phase protection protein Dps	+2.88	5.77E-04
	BHW77_01455	*uspB*	universal stress protein UspB	+3.07	1.96E-03
	BHW77_14630	*uspF*	universal stress protein F	+2.01	3.42E-04
	BHW77_20660	*uspG*	universal stress protein UspG	+2.76	3.62E-04
	BHW77_18490	*hspQ*	heat-shock protein HspQ	+2.29	1.12E-03
	BHW77_00060	*ibpA*	heat-shock protein	+2.15	2.84E-02
	BHW77_10075	*cspG*	cold-shock protein	+2.99	2.31E-06
	BHW77_06270	*clpB*	ATP-dependent chaperone ClpB	+2.72	3.11E-03
	BHW77_02235	*fic-1*	cell filamentation protein	+2.95	8.37E-04
	BHW77_18535	*sulA*	cell division inhibitor	+1.80	8.47E-04
	BHW77_19955	*cpoB*	cell division protein	−2.61	2.01E-07
	BHW77_18715	*mukB*	cell division protein	−2.12	6.72E-08
	BHW77_14120	*dosC*	diguanylate cyclase	+2.87	3.86E-08
	BHW77_21465	*bolA*	transcriptional regulator	+3.22	2.57E-05
	BHW77_19190	*bssR*	transcriptional regulator	+2.76	1.25E-04
	BHW77_11525	*bssS*	transcriptional regulator	+7.75	4.94E-08
	BHW77_17570	*flhC*	transcriptional regulator	−1.62	2.44E-02
	BHW77_16255	*sodC*	superoxide dismutase	+ 2.81	3.37E-04

^a^Gene group/gene designations were selected from RAST Server [109]

^b^Gene name and known or predicted functions are based on the annotated sequence of *E. coli* O157:H7 EDL 933 [110]

^c^ + Symbol represents that the gene expression listed in the column was upregulated

### Norepinephrine enhanced expression of genes encoding acid resistance and signaling system EvgS/EvgA

**Fig. 2:**
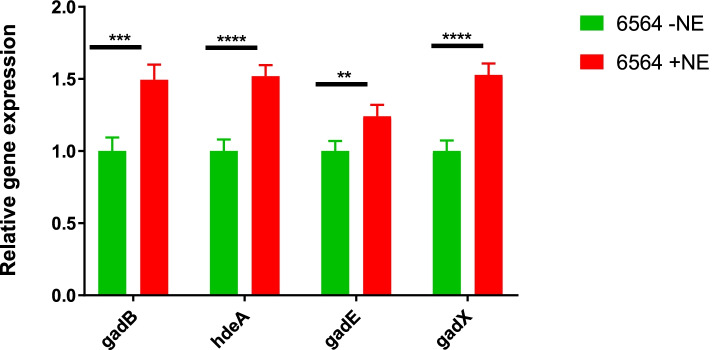
Determination of expression of genes encoding acid resistance pathway 2. Relative expression of *gadB, hdeA, gadE* and *gadX* genes was determined by using total RNA prepared from strain NADC 6564 grown without (green bars) or with norepinephrine (red bars). Error bars represent standard deviation of the mean of three independent assays. *** *p* = 0.00043, **** *p* = 0.000086; ** = *p* = 0.0037; **** *p* 0.000064

O157 can survive for several hours in highly acidic environments (pH 2.0 to 2.5) resembling those encountered in a mammalian stomach [46]. Similar to the results reported in a previous microarray-based transcriptional study [34], we also observed that NE induced significantly higher (≥ 2.0-fold, *p* < 0.05) expression of acid resistance pathway (ARP2) genes involved in O157 strains’ extreme acid tolerance (Table 3 and Table S2). The highly (*p* < 0.05) induced ARP2 genes included *gadA* (+ 4.21-fold), *gadB* (+ 4.36-fold), and *gadC* (+ 5.02-fold), which encode enzymes for reducing cytoplasmic H^+^ ion concentration when external pH is very acidic. Similarly, expression of *hdeD*, *hdeB*, and *hdeA*, which are present on an acid fitness island and encode proteins that serve as chaperons during acid stress [47], was upregulated by + 4.76, + 3.46, and + 3.94 -fold, respectively (Table 3, Table S2). The *gadE* gene, a LuxR-like family of proteins and a master regulator of ARP2 genes [48], was significantly upregulated (+ 3.72-fold, *p* < 0.05) in NE-treated cultures. The expression of *gadX* (+ 3.27-fold) and *gadW* (+ 2.37-fold), which encode AraC-family of transcriptional regulators GadX and GadW [20], respectively, and control *gadA/BC* and *gadE* expression, was significantly upregulated in NE-treated cultures. We also used RT-qPCR to determine the relative expression of some of the genes (*gadB*, *hdeA*, *gadE*, and *gadX*) of ARP2 that showed elevated expression by RNA-Seq. Although RNA-Seq analysis showed an increase of ≥2.0 -fold in the expression of *gadB, hdeA, gadE,* and *gadX* in NE-treated cultures, RT-qPCR analysis showed significant increases in the expression of these genes but the fold increase in their expression was < 2-fold (Fig. 2) (Table 3; Table S2). The most important reason for the observed differences in gene expression by these two approaches could be that RNA-Seq libraries were normalized and gene expression values were calculated relative to the mRNA pool. In RT-qPCR, the amount of RNA used for cDNA synthesis was based on total RNA levels, which could lead to variability in RNA available for cDNA synthesis necessitating the use of an internal reference for normalization. But despite these underlying technical differences, the trend showing upregulation of four ARP2 genes was similar between the two methods. Similar findings were apparent in a study where use of a RT-qPCR as validation approach produced lower fold changes in the expression of genes compared to that detected for the same genes by microarray-based transcriptional analysis [34].

**Table 3:** Norepinephrine enhanced expression of genes linked to acid resistance pathway 2 and *evgS/evgA* encoding a two-component signal transduction system

Pathway/Gene group^**a**^	Locus tag	Gene^**b**^	Gene function	Fold change^**c**^	***p*** adjusted
**Acid resistance/Acid resistance pathway 2 (ARP2)**					
	BHW77_01285	*gadA*	glutamate decarboxylase	+ 4.21	1.61E-03
	BHW77_01290	*gadX*	transcriptional regulator	+ 3.27	3.18E-07
	BHW77_01295	*gadW*	AraC family transcriptional regulator	+ 2.37	7.84E-04
	BHW77_01315	*gadE*	transcriptional regulator	+ 3.72	5.69E-07
	BHW77_01320	*hdeD*	protein	+ 4.76	9.22E-05
	BHW77_01325	*hdeA*	acid stress chaperone	+ 3.46	9.30E-05
	BHW77_01330	*hdeB*	acid stress chaperone	+ 3.94	8.70E-07
	BHW77_14105	*gadB*	glutamate decarboxylase	+ 4.36	1.09E-03
	BHW77_14110	*gadC*	glutamate:gamma-aminobutyrate antiporter	+ 5.02	6.32E-04
	BHW77_07400	*evgS*	two-component system sensor histidine kinase	+ 2.10	5.37E-05
	BHW77_07405	*evgA*	DNA-binding response regulator	+ 2.93	9.85E-21

^a^Gene group/gene designations were selected from RAST Server [109]

^b^Gene name and known or predicted functions are based on the annotated sequence of *E. coli* O157:H7 EDL 933 [110]

^c^ + Symbol represents that the gene expression listed in the column was upregulated

**Fig. 3:**
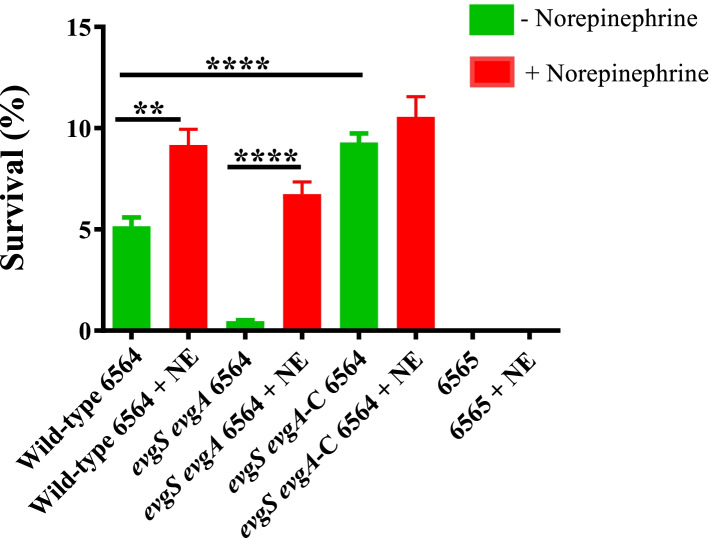
Determination of the requirement of *evgS/evgA* genes in survival of *E. coli* O157:H7 strain NADC6564 in highly acidic medium and in NE signaling. The overnight bacterial cultures of parental strain NADC 6564 carrying the cloning vector pACYC177 (NADC 6564/pACYC177), *evgS/evgA* deletion mutant of NADC 6564 carrying pACYC177 (strain NADC 6662/pACYC177), *evgS/evgA* deletion mutant complemented with *evgS/evgA* recombinant plasmid pSM779 (strain NADC 6662/pSM779), and an acid sensitive strain of *E. coli* O157:H7 carrying pACYC177 (strain NADC 6565/pACYC177) were grown in DMEM medium lacking (green bars) or containing norepinephrine (red bars) were diluted in a phosphate-citrate minimal medium acidified to pH 2.5. After 3 h of incubation, cultures were plated for viable cell count determination (as described in materials and methods) Error bars represent standard deviation of the mean of three independent assays. **** *p* < 0.0001, * *p* = 0.033; ** = *p* = 0.0086

In addition to the upregulation of *gadE, gadX,* and *gadW,* NE enhanced expression of *evgS* (+ 2.1-fold; *p* < 0.05) and *evgA* (+ 2.93-fold; *p* < 0.05) (Table 3; Table S2). The EvgS/EvgA signaling system is involved in the regulation of ARP2 in *E. coli* through YdeO, the transcriptional factor that activates *gadE* [24]. However, *ydeO* expression was not enhanced in NE-treated cultures suggesting that EvgS/EvgA might activate *gadE* directly without the intermediate of YdeO. To confirm a direct requirement of EvgS/EvgA in ARP2 expression and NE signaling, we constructed an *evgS/evgA* deletion mutant of strain NADC 6564 and compared the *evgS/evgA* mutant and the same mutant complemented with an *evgS/evgA* recombinant plasmid to the parental strain NADC 6564 in their ability to survive exposure to highly acidic (pH 2.5) conditions before or after exposure to NE*.* As shown in Fig. 3, the *evgS/evgA* mutant grown overnight without NE and then incubated for 3 h in phosphate-citrate minimal medium (pH 2.5) was recovered at significantly lower numbers (0.47%, *p* < 0.05) compared to 5.16 and 9.3% recovery of viable cells of parental strain NADC 6564 and the *evgS/evgA* mutant complemented with an *evgS/evgA*-recombinant plasmid, respectively. On the other hand, the *evgS/evgA* mutant grown overnight in the presence of NE and then exposed to an acidified medium for 3 h resulted in the recovery of significantly higher numbers of viable cells (6.74%, *p* < 0.05) compared to the mutant strain (0.47%) grown overnight in the absence of NE (Fig. 3). However, recovered numbers of viable cells for the *evgS*/*evgA* mutant even after an overnight growth in NE were still lower (6.74%, *p* < 0.05) compared to the similarly grown cultures of parental strain (9.17%) and the complemented *evgS/evgA* mutant strain (10.55%) (Fig. 3). These results indicated that *evgS*/*evgA* genes are involved in ARP2-mediated acid resistance of strain NADC 6564 as mutants lacking these genes were highly sensitive to very low pH conditions. However, the *evgS*/*evgA* mutant was still able to respond to NE signaling but at levels that were only slightly lower in terms of recovery of viable cells (6.74%, *p* = 0.03) after 3 h exposure to acidic medium than the similarly grown parental strain (9.17%) (Fig. 3).

### Norepinephrine enhanced expression of genes encoding various stress response, cell division, and biofilm formation pathways

In addition to enhancing expression of ARP2 genes, the presence of NE resulted in the differential expression of genes linked to various stress responses. Prominent among these genes were those that enable *E. coli* strains to survive in the stationary phase of growth. As listed in Table 2 and Table S2, stationary phase genes that were significantly (≥ 2.0-fold, *p* < 0.05) upregulated in NE-treated cultures was the DNA starvation/stationary phase protection gene *dps* (+ 2.88-fold) [49] and genes *uspA, uspB, uspE, uspF,* and *uspG*, which encode universal stress proteins [50]. The expression of *usp* genes was upregulated in the range + 2.01-fold through + 3.07-fold in response to NE. Another set of genes that was upregulated in response to NE is an important component of heat shock response in *E. coli* [51]. Some of these genes included *hspQ* (+ 2.2-fold) and *ibpA* (+ 2.15-fold) (Table 2, Table S2). The heat shock response is initiated in response to a sudden increase in the growth temperature while the cold shock proteins (Csp) are produced in response to rapid temperature downshifts [52]. As shown in Table 2 and Table S2, *cspG* expression was significantly upregulated (+ 2.99-fold) in response to NE. Also, upregulated was *clpB* (+ 2.72-fold) encoding a protease produced during stress [52]. The *sodC* gene that encodes a periplasmic superoxide dismutase C in NE-treated cultures (Table 2) and serves as an important antioxidant in protecting bacterial cells from oxidative stress [53–56] was also upregulated by + 2.81-fold in response to NE.

Since unfavorable growth conditions promote biofilm formation that requires the induction of many stationary phase-dependent genes [57, 58], NE-treatment induced significantly (*p* < 0.05) higher expression of some of the genes linked to biofilm formation. Prominent among these genes was *csgB* (+ 7.85-fold) (Table 1 and Table S2) that encodes the CsgB protein essential for the formation of curli fimbriae. Curli fimbriae are essential for the initial, reversible bacterial adherence to abiotic/biotic surfaces and in subsequent stages of biofilm formation [59]. The expression of genes (*wcaA* and *wcaB*) encoding enzymes for the biosynthesis of extracellular polysaccharide colanic acid was significantly downregulated (≥ − 2.0-fold, *p* < 0.05) in response to NE (Table S3). Other genes that were upregulated in response to NE have been implicated in the control of biofilm formation including *bolA* (+ 3.2-fold), *bssR* (+ 2.76-fold), and *bssS* (+ 7.75-fold) (Table 2 and Table S2). The *bolA* gene confers round cell morphology to *E. coli* cells, is expressed in the stationary phase of growth in a RpoS-dependent manner, and controls biofilm formation in *E. coli* [60, 61]. The *bssR* (yliH) and *bssS* (yceP) genes encode transcriptional regulators involved in the regulation of biofilm formation through autoinducer-2 secretion in *E. coli* K12 [62].

### NE-treatment resulted in differential expression of genes encoding metabolic pathways

Table 4 lists the numbers of genes differentially expressed in various metabolic pathways in response to NE. Tables S2 and S3 list names of differentially expressed genes (≥ 2.0-fold, *p* < 0.05), pathways represented by these genes, and fold-change in the expression of these genes. Among the metabolic pathways that had their representative genes significantly upregulated were ABC transporter systems for uptake of amino acids, glutamine, and sulfate/sulfite (Table 4; Table S2). Also upregulated were genes encoding phosphofructokinase *fruK* (+ 3.12-fold, *p* < 0.05) and PTS fructose transporter subunit IIBC (+ 4.32, *p* < 0.05) (Table 4; Table S2), suggesting that gluconeogenic activity might be enhanced in response to NE. The genes *mglA* (− 4.26-fold, *p* < 0.05) and *mglC* (− 2.66-fold, *p* < 0.05) encoding galactose/methyl galactoside ABC transporter ATP-binding protein and galactoside ABC transporter permease, respectively, were significantly downregulated suggesting that the transport of readily metabolizable sugars was reduced in NE-treated cultures (Table 4 and Table S3). The other downregulated (≥ 2.0-fold, *p* < 0.05) genes included *livG* and *livM* encoding a high-affinity branched-chain amino acid ABC transporter ATP-binding protein LivG and high-affinity branched-chain amino acid ABC transporter permease LivM, respectively; *dppB*, *dppC*, *dppD* and *dppF* encoding dipeptide ABC transport system; *cstA* encoding an inner membrane peptide transporter [63]; and genes *pstA*, *pstB*, *pstC* and *pstS* encoding uptake system for inorganic phosphate, which is required for phosphorylation of cellular proteins, lipids, and carbohydrates (Table S3). The other upregulated (≥ 2.0-fold, *p* < 0.05) genes were those that encoded the pyrimidine utilization pathway for assimilating pyrimidine as a sole nitrogen source [64]. Also significantly upregulated were genes encoding nitrate/nitrite transport and nitrite/nitrate reductases that oxidize nitrite and nitrate, respectively, as terminal electron acceptors during anaerobic metabolism [65] (Table 4; Table S2). With respect to iron transport, the expression of *feoABC* genes, which are involved in ferrous iron uptake system, was significantly upregulated, but the expression of genes for ferric iron uptake system (*fepC* and *fepG*) and genes (*entA*, *entC*, *entE*) for the biosynthesis of siderophore enterobactin were downregulated by ≥2.0-fold (Table 4 and Table S2 and Table S3). On the other hand, genes *cirA* and *fiu* encoding catecholate siderophore (enterobactin) receptor proteins were significantly downregulated. The expression of many genes representing pathways for transcription, translation, and energy production was also significantly downregulated in response to NE (Table 4 and Table S3). These downregulated genes included *rpoA* and *rpoB*, which encode DNA-directed RNA polymerase subunit α and β, respectively; 11 and 15 genes encoding 30S and 50S ribosomal proteins essential for protein synthesis; two genes (*trmG* and *tgt*) involved in tRNA modification; and genes *fus, tsf*, *tuf* encoding translational elongation factors G, Ts, and Tu. For the energy generating pathways, the genes encoding for cytochrome b, c, and o; electron transport complex subunit RsxE and RsxG; and subunits α, β, γ, δ and ε of F0F1 ATP synthase required for the synthesis of ATP [66] were also significantly downregulated (Table 4 and Table S3). In addition, expression of many genes encoding enzymes involved in carbohydrate, amino acid, and fatty acid metabolism was also significantly downregulated in NE-treated cultures (Table 4; Table S3). The representative genes of these pathways were *glpT*, which encodes a transporter for the uptake of glycerol-3-phosphate used as a substrate in glycolysis and phospholipid biosynthesis [67]; *sucA* encoding 2-oxoglutarate dehydrogenase E1 component for converting 2-oxoglutarate to succinyl-CoA and CO_2_ in the TCA cycle; *sdhCDAB* encoding a succinate dehydrogenase enzyme complex for synthesizing fumarate from succinate; genes encoding NADH-quinone oxidoreductase that serve as a main entry point for electron transfer to the electron transport chain to generate ATP [68]; *purCDFLMNT* catalyzing de novo purine biosynthesis; and *fadB* that encodes a multifunctional fatty acid oxidation complex subunit alpha for aerobic and anaerobic degradation of long-chain fatty acids. Also downregulated was the expression of genes *tnaA* (+ 2.87-fold) and *tnaL* (+ 6.99-fold) required for the hydrolysis of tryptophan to produce indole that plays an important role in the regulation of biofilm formation [69] (Table 4 and Table S3). Besides inducing differential expression of genes encoding various metabolic pathways, NE also impacted expression of genes involved in bacterial cell division. Some of these genes, such as *cpoB*, which coordinates cell wall production and the movement of the outer membrane during cell division [70], and *mukB*, which is essential for the correct partitioning of replicated chromosomes during cell division so that both daughter cells inherit a copy of the replicated chromosome [71], were significantly downregulated (≥ − 2.00-fold, *p* < 0.05) (Table 2 and Table S3). On the other hand, the expression of *fic-1*, whose function is not fully understood but impacts cell division by leading to cell filamentation [72], was significantly upregulated (+ 2.95-fold) in response to NE (Table 2 and Table S2). Strain NADC 6564, like other *E. coli* O157:H7 strains, harbors a cluster of genes (*terABCWZ* and *tehB*) encoding resistance to tellurium. The expression of tellurium resistance genes that might be involved in bacterial resistance to host cellular defenses [73] was also downregulated (≥ − 2.0-fold, *p* < 0.05) in response to NE (Table 4 and Table S3).

**Table 4:** Differential expression of various metabolic pathways in response to norepinephrine

Upregulated pathways^a^		Downregulated pathways^a^	
Pathway^b^	Number of genes with increased expression^b^	Pathway^b^	Number of genes with decreased expression^b^
Amino acid ABC transport	3	3’,5’-cyclic-AMP phosphodiesterase	1
Amino acid transport	2	30S ribosomal proteins	11
23S and 16S rRNA methylation	4	50S ribosomal proteins	17
Anaerobic ribonucleotide reductase and dehydrogenase	2	6-phosphofructokinase II	1
Cell division/cell division inhibition	4/1	ABC transporter ATP-binding protein	2
Cytochrome oxidase	1	ABC transporter permease	2
Diguanylate cyclase/phosphoesterase	1/1	Acetate-CoA ligase	1
DNA replication, modification and repair	7	Acetolactate synthase	4
DUF domain containing proteins	21	Acetyl-CoA transferase/carboxylase	1/3
Ferrous ion transport	3	Adenine uptake/utilization	5
Glutamate metabolism	5	Alanine utilization	4
Glutamine ABC transport	3	Alcohol/Aldehyde dehydrogenase	1/1
Glycogen synthesis	1	Aldolase	1
Hypothetical proteins	87	Aspartate metabolism	6
Integrases/transposases	8/12	ATP F0F1 synthase subunit C	
LPS biosynthesis	4	C4-dicarboxylate transporter	
Multidrug ABC transport/efflux	2/2	Bifunctional aspartate kinase, cysteine desulfurase, glutamine synthase	3
Multidrug transport	6	Enterobactin biosynthesis	2
NAD(P)-dependent oxidoreductases	3	Cell division proteins	8
Nitrate/nitrite transport	3	Citrate metabolism	2
Nitrate/nitrite reductases	2/2	Cytochrome b	2
Outer membrane proteins	4	Cytochrome c biogenesis protein	2
Oxidoreductases	6	Cytochrome O ubiquinol oxidase	4
Pyrimidine utilization proteins	5	Cytosine metabolism	2
Sugar fermentation stimulation protein	1	Dipeptide ABC transport	4
Sugar transporter	1	Electron transport complex subunit RsxD, E, and G	3
Sulfate/sulfite transport metabolism	9	F0F1 ATP synthase	7
Zn transport PTS fructose transporter	2 1	Fe2+ -enterobactin and Fe^3+^-hydroxamate ABC transport proteins	2
		Ferredoxin reductase	2
		Fe-S cluster assembly proteins	6
		Formate dehydrogenase	5
		Fructose biphosphate	1
		Galactose metabolism	3
		Glucan biosynthesis	2
		Glutathione metabolism	6
		Glycerol metabolism	4
		Glycine metabolism	5
		Heme/hemen transport and utilization	4
		Branched-chain amino acid transport	2
		Hypothetical proteins	101
		Iron ABC transporter	1
		Iron-enterobactin and iron-hydroxamate transport	4
		Isochorismate/isochorismate synthase EntC	2
		LPS biosynthesis	3
		Microcin transport	3
		Molybdate transport and molybdenum cofactor biosynthesis	6
		Multidrug transporter MdtBCDJ	4
		Multifunctional acyl-CoA and fatty acid oxidation complex	3
		Murein biosynthesis	9
		NAD(P)-dependent, NAD, NADH and NADHP- dependent enzymes	14
		Nitric oxide dioxygenase and reducatse	2
		Oxidative damage protection protein/defense proteins	1/1
		Peptide ABC transport	2
		Peptidylprolyl isomerases (proper protein -folding)	5
		Phage shock proteins (PspBDA)	3
		Phosphate transport	6
		Phosphoenolpyruvate--protein phosphotransferase	2
		phosphoethanolamine transferase	1
		phosphoribosylamine-glycine ligase and 5 other enzymes of purine biosynthesis	6
		Preprotein translocase subunits SecY, SecA, YajC, YidC	4
		Protein-export membrane protein SecD, SecF	
		PTS glucose/sugar transporter	1/1
		Putrescine/Spermidine ABC transporters	1/1
		pyruvate dehydrogenase complex dihydrolipoyllysine-residue acetyltransferase	1
		Pyruvate Kinase	2
		Serine metabolism	2
		Spermidine/putrescine metabolism	2
		Succinate dehydrogenase	7
		Tellurite resistance proteins	7
		Thiamine ABC transport	2
		Threonine synthase	1
		Thymidylate synthase	1
		Transcriptional regulator FlhC	1
		Translation elongation factors G, Ts, Tu, Tu, IF-2, IF-3	6
		Transposase	2
		tRNA modification enzyme complexes	11
		Tryptophanase	1
		Type II secretion system	6
		Tyrosine-protein kinase	1
		UDP-N-acetylglucosamine and UDP-N-acetylmuramate-amino acid ligases (cell wall biosynthesis)	9
		Urease accessory proteins and subunits α, β, γ	7

^a^ Pathway/gene names their known or predicted functions are based on the annotated sequence of *E. coli* O157:H7 EDL 933 [110]

^b^ Detailed description of enzymes/proteins encoded by genes differentially expressed at ≥ 2-fold and representing different pathways is given in Supplementary Tables S2 and S3

### NE had no effect on growth rate but enhanced viability

Although the bacterial growth curves generated over a 24 h of growth in the presence or absence of NE were almost identical (Fig. S1A), viable counts recovery was about 2-fold higher in NE-treated cultures compared to the culture grown without NE (Fig. S1B).

## Discussion

The RNA-Seq-based transcriptional profiling of *E. coli* O157:H7 strain NADC 6564 [43] grown in the presence of norepinephrine (NE) to an early stationary phase showed differential expression (DE) of large number of genes that are usually turned on in the stationary phase plus genes involved in bacterial virulence, stress response, and various metabolic pathways. The pattern of differential expression for many of these genes was highly similar to that reported for these genes by a microarray-based approach in *E. coli* O157:H7 (O157) strain EDL933 [34]. Despite differential expression of many related genes in response to NE between EDL933 and NADC 6564 strains, which are classified as lineage I strains [74], these two strains differ in having distinct regulatory systems controlling expression of virulence genes. The RNA-Seq-based approach identified many differentially expressed genes in strain NADC6564 that were not represented in a microarray-based transcriptional profile of strain EDL933, since that study only probed 610 genes for DE in response to NE [34]. For example, NE exposure not only enhanced the expression of genes encoding (*gadABC*) and controlling (*gadE, gadX,* and *gadW*) glutamate-dependent resistance pathway 2 (ARP2) [15, 19, 20] similar to that was observed for strain EDL933 [34], but also upregulated genes encoding the EvgS/EvgA signaling system implicated in the regulation of ARP2 in *E. coli* [22, 75]. We validated this finding for the first time by demonstrating that deleting *evgS/evgA* genes reduced resistance of strain NADC 6564 to highly acidic environment but the NE signaling was not dependent on EvgS/EvgA.

Similar to previous findings using EDL933 strain [34], the expression of several LEE genes was significantly increased in strain NADC 6564 in response to NE, but the expression of *ler*, which encodes Ler for activating LEE expression, was not significantly altered with or without exposure to NE. We have shown previously that the basal level of *ler* transcription is very high in strain NADC 6564 [76], and in the current study we found that the growth of this strain in the presence of NE had no significant effect on *ler* expression (− 1.40-fold, *p* = 0.311, Table S1). Both LEE- and non-LEE-encoded transcriptional factors, such as RpoS, QseA, PerC, Hha, IHF, SdiA, H-NS, Fis, GrlA, GrlR, GadE, GadX, RcsB, and Hfq control *ler* expression by their direct interactions with *ler* promoter region [10, 77]. For example, sRNA chaperone Hfq acts as a negative regulator of LEE in EDL933 but in strain NADC 6564 LEE expression is positively regulated by Hfq [78]. Similarly, RpoS, the stationary sigma factor, directly or in conjunction with other regulators, can have positive or negative regulatory effect on LEE expression [79, 80]. However, NE exposure had no significant effect on Hfq and RpoS expression in strain NADC 6564 suggesting that LEE activation in response to NE occurs via a different regulatory mechanism. The transcriptional factors that were upregulated in response to NE in strain NADC 6564 were GadE, GadX, and 2 of the 4 copies of *perC*, which encode transcriptional factor PerC. Despite the upregulation of *gadE* and *gadX*, which have been shown to repress LEE gene expression [10, 77], LEE expression was upregulated in response to NE. Thus, it is possible that upregulated PerC family of proteins, which have been shown to increase LEE expression by interacting with the *ler* promoter [81], could account for the upregulation of LEE gene expression by inhibiting GadE- and GadX-mediated repression of *ler*. Besides LEE-encoded virulence adherence proteins, *E. coli* O157:H7 strains can express different types of fimbriae in response to a variety of intestinal metabolites, such as ethanolamine, choline, and serine [82]. In the RNA-Seq data, we also observed significantly increased expression of fimbrial genes belonging to Lpf1 and Ybp fimbrial groups in response to NE. The Lpf1 fimbriae have been shown to promote bacterial adherence to epithelial cells and mutants lacking genes encoding Lpf1 fimbriae show poor colonization in animal models [83, 84]. Since expression of these fimbriae is modulated in vitro both by environmental factors and bacterial- and host-produced metabolites [82, 84], presence of NE in mammalian intestine at concentrations sufficient to induce differential gene expression could promote expression of the above listed fimbriae leading to increased adherence of *E. coli* O157:H7 to host tissues.

Transcriptional profiling in response to NE revealed differential expression of many stress response genes that are usually turned on as bacterial cells enter a stationary growth phase or encounter conditions less optimal for growth. Some of these genes, which were also differentially expressed in EDL933 using the microarray approach [34], were represented by *usp* genes that encode Usp superfamily of proteins performing a diverse array of functions related to oxidative stress, iron homeostasis, motility/adhesion, which could impact pathogenesis of O157 strains [50, 85, 86]. Similarly, the significant induction of heat and cold shock response genes in NE-treated cultures would be important in maintaining protein homeostasis by assisting in the folding of newly synthesized proteins, preventing protein aggregation, rescuing partially or completely un-folded proteins formed under stress, and preventing the formation of secondary structures in mRNA at low temperatures to allow the initiation of translation [51, 52]. In addition, we also observed upregulation of genes *dps* and *clpB* that are important in preventing oxidative damage to DNA from hydrogen peroxide produced during specific metabolic activities and removal of damaged polypeptides from stressed bacterial cells, respectively [49, 52].

Since biofilm formation is induced under conditions unfavorable for growth, such as when bacterial cells enter the stationary phase, experience nutritional stress, or low temperatures [59], several genes linked directly or indirectly to biofilm formation were differentially expressed by NE. Among the genes that are directly linked to biofilm formation, *csgB* was highly upregulated in response to NE. The CsgB protein facilitates assembly of CsgA, a major curli subunit into mature curli fimbriae, which are essential for the initial bacterial adherence to abiotic/biotic surfaces during biofilm formation [57, 59, 87, 88]. The genes *csgB* and *csgA* constitute, along with *csgC,* the *csgBAC* operon transcribed divergently from the *csgDEFG* operon [44]. The expression of genes in *csgBAC* and *csgDEFG* opeons is positively regulated by the global transcriptional factor CsgD encoded by the *csgD* gene of *csgDEFG* operon [89, 90]. In addition, the *csgEFG* gene products are essential in the secretion and assembly of CsgA in to curli fimbriae [59, 91] and CsgD regulates expression of other genes, such as those encoding bacterial cellulose that are essential for biofilm formation [92]. Despite the upregulation of *csgB* and presumably other genes in this operon, we did not detect any change in the expression of *csgD* or the *csgEFG* genes when cultured with or without NE. These results were corroborated by the inability of strain NADC 6564 to produce biofilms when grown with or without NE for 72 h (data not shown) according to a previously described biofilm detection procedure [93]. The apparent lack of any increase in *csgD* expression could be attributed to the inability of NE to cause any changes in the differential expression of *rpoS, rcsB, fis* and *hha* genes, which have been shown to play important role in biofilm formation by O157 by affecting *csgD* expression [93–95]. Since increased expression of *perC* homologs has been shown to repress *csgD* expression and biofilm formation [96], it is also possible that increased expression of two of the four copies of *perC* homologs in response to NE could have resulted in *csgD* repression leading to no increases in biofilm formation by strain NADC 6564.

Besides upregulation of pathways impacting bacterial virulence and response to various stressors, large number of genes encoding a variety of metabolic pathways were differentially impacted in their expression by NE. The majority of upregulated genes were those that enabled *E. coli* O157:H7 to utilize alternative sources of carbon and nitrogen, such as amino acids and pyrimidines, rather than the readily utilizable sugars that probably are scarce in the stationary phase-like growth conditions and in the host intestinal environment. There was also significant upregulation of nitrate/nitrite transport and nitrite/nitrate reductases that oxidize nitrite and nitrate as terminal electron acceptors in anaerobic metabolism [65]. Additional support that the metabolism of NE-treated cultures become less aerobic is garnered by the increased expression of fumarate reductase, which is a terminal electron receptor in fermentative metabolism of carbon substrates [97]. Thus, this shift to less aerobic metabolic activity in response to NE may account for the downregulation of other prominent metabolic pathways such as glycolysis, TCA cycle, electron transport system for producing ATP, fatty acid oxidation, gene transcription, and protein synthesis. This altered metabolic physiology and differential upregulation of genes promoting adherence to tissues and resistance to various stressors might also be correlated to differential expression of genes that inhibit cell division (*cpoB*, *mukB*, and *fic-1*) and alter cellular morphology (*bolA*). It has been suggested that altered cellular morphology during stationary phase might be a strategy to tolerate variety of stresses and nutritional starvation [98]. Although, some studies have reported that the exposure of *E. coli* O157:H7 strains to NE for 4 to 6 h can increase growth rate by a 1/100 of an A_600_ [34], we didn’t detect such a small change in growth of strain NADC 6564 grown in minimal medium containing NE relative to that grown without NE. However, a higher number of viable cells were recovered from cultures grown in the presence of NE, suggesting that altered metabolic profile, and differential expression of many stress-related, and stationary phase-dependent pathways might enhance survival and host colonization potential of O157 strains when exposed to NE during the stationary phase-like growth conditions [27, 98–100].

## Conclusions

Based on the whole genome transcriptional profiling of *E. coli* O157:H7 strain NADC 6564 grown in the presence of NE to an early stationary growth phase, we observed that NE exposure had a major impact on the expression of genes attributable to bacterial survival under suboptimal growth conditions, such as those encountered during stationary phase of growth, during colonization of the host intestinal mucosa, and during bacterial persistence in the environment outside of the host animal. Thus, the availability of NE and other host-produced metabolites could serve as signals and/or nutrients to not only alter the global gene expression profile but also skew the gene expression profile to the benefit of *E. coli* O157:H7 by enhancing its ability to colonize the carrier host animal, produce disease in the susceptible human host, and survival outside the host animal.

## Materials and methods

### Bacterial strains and growth conditions

Bacterial strains used in this study are listed in Table 5. *Escherichia coli* O157:H7 strain NADC 6564 served as the parental strain and all other strains were derivatives of this strain, either described previously or in the current study. *E. coli* TOP10 was used as a host for the propagation of recombinant plasmids. Bacterial strains were propagated in Luria-Bertani broth (LB) or LB containing 1.5% agar (LB-agar). Antibiotics were added to liquid or solid media as needed (streptomycin 100 mg per liter; carbenicillin 100 mg per liter; kanamycin 50 mg per liter).

**Table 5:** Bacterial strains and plasmids

Strain or plasmid^**a**^	Genotype and description	Source or reference
***E. coli*** **strains**		
NADC 6564	*stx2*^*+*^ and streptomycin-resistant *E. coli* O157:H7	[43]
NADC 6565	Acid-sensitive *rcsB* mutant strain of NADC	[94]
NADC 6662	*evgS evgA* deletion mutant of NADC 6564	This study
TOP 10	F^-^ *mcrA* Δ(*mrr-hsd*RMS-*mcr*BC) Φ80*lacZ*ΔM15	Life Technologies
	Δ*lac*X74 *rec*A1 *ara*D139 Δ(*ara*-*leu*)7697 *gal*U	
	*gal*K *rpsL* (Str^R^) *end*A1 *nup*G	
**Plasmids**		
pACYC177	Low-copy cloning vector	New England Biolabs
pKD46	Recombineering vector	[106]
pCP20	FLP recombinase vector	[106]
pSM779	4.17 kb *evgS evgA* operon isolated by PCR from strain NADC 6564 and cloned at *Sma*I site of pACYC177	This study

^a^ Detailed description of the construction of bacterial strains and plasmids listed are provided under material and methods

### Transcriptional profiling

For RNA isolation, an overnight bacterial culture grown at 37 °C in LB broth was diluted 1:100 (A_600_ = 0.10) into a low-glucose Dulbecco’s Minimal Eagles Medium (DMEM) lacking or containing 50 μM norepinephrine, the amount considered to be reached locally in various areas of GIT [101]. After about 5.5 h of incubation at 37 °C with shaking (250 rpm) to allow cultures to attain A_600_ ≈ 1.2, (the incubation period which we and others have shown in a previous study to be long enough to allow bacterial cultures to reach the early stationary phase of growth [34, 76]), total RNA was isolated using RNeasy isolation kit according to the manufacturer’s instructions (Qiagen, Valencia, CA). RNA was treated with DNase (TURBO DNA-free kit; ThermoFisher Scientific, Grand Island, NY). The DNase-treated RNA was used for RT-qPCR or treated with Ribo-Zero rRNA kit reagents according to the manufacturer’s instructions (Gram-negative bacteria; Illumina, Inc., San Diego, CA) to remove rRNA. The strand-specific RNA-Seq libraries were prepared from the rRNA-free RNA and sequenced with Illumina HiSeq (Iowa State University, Ames, Iowa). The trimmed, single-end reads were mapped to the reference genome (strain NADC 6564) using HiSat2 v2.05 to generate SAM files that were fed into htseq-count v0.11.2 along with the reference genome file for unnormalized read quantification. DESeq2 was used to determine differential gene expression by analyzing quantified htseq-counts. The set of differentially expressed genes for each comparison were sorted by the adjusted *p*-value of less than 0.05. A total of three biological replicates of bacterial cultures grown independently were used for RNA-Seq analysis.

### Read QC and mapping

The trimmed reads acquired from sequencing were first run through FastQC v0.11.5 to check for any glaring issues in the quality of reads. After it had been determined that there weren’t any major discrepancies associated with the reads, the single-end reads were mapped to the reference genome using HiSat2 v2.05. HiSat2 utilizes a novel indexing scheme termed Hierarchical Graph FM index which improves the efficiency of pattern recognition [102]. First, HiSat2 was provided with the reference FASTA file for *E. coli* O157:H7 strain NADC 6564 acquired from the NCBI database [43, 103]. The reference FASTA file was indexed by HiSat2 to make mapping possible using HiSat2 algorithms. Once the HiSat2 index for NADC 6564 had been built, the trimmed FASTQ files for all NADC 6564 control and norepinephrine-treated replicates were passed as input to HiSat2. Other than multiple threads being used to speed up the processing time, default HiSat2 parameters were used to conduct the mapping. The output SAM file for each replicate was used as an input for read quantification.

### Read quantification

The mapped reads were fed into htseq-count v0.11.2 along with the reference GFF file for unnormalized read quantification [103]. The GFF annotation file was acquired from the same NCBI accession for *E. coli* O157:H7 strain NADC 6564 as mentioned previously. The stranded option was specified as “no” because a strand-specific sequencing protocol was not used. The default “union” overlap resolution mode was used in order to avoid discriminating valid reads [103]. The “CDS” tag was used as the feature type to quantify. The output text file was formatted for the next step by removing the quantification statistics at the end and adding column names.

### Differential gene expression analysis

DESeq2 was used to identify differentially expressed genes in the data set [104]. To do so, DESeq2 was launched in an R environment and the quantified reads were imported as a tab-delimited table. An experimental design table specifying control and experimental groups was created according to the format specified by the DESeq2 vignette. Once the quantified read data and experimental design was provided, DESeq2 was run with the default false detection rate set to 0.1. The genes were tested using the default null hypothesis of not being different. Gene expression was compared between the control and treated strain. The set of differentially expressed genes were sorted by the adjusted *p*-value of less than 0.05.

### Pathway analysis

The resultant set of differentially expressed genes were mapped to genes in *E. coli* O157:H7 str. EDL933. The corresponding differentially expressed genes in EDL933 were used as input to STRING-DB. STRING-DB is a database of known and predicted protein-protein interactions that was used to search for correlations between the gene products that were found to be differentially expressed [105]. Images characterizing the network of protein-protein interactions between the differentially expressed products were created by STRING-DB. In addition, KEGG annotation for the differentially expressed genes are also provided by STRING-DB.

### Recombinant DNA procedures

The *evgS/evgA* deletion mutant of *E. coli* O157:H7 strain NADC 6564 was constructed by using a phage lambda-derived Red recombination system [106]. Briefly, a 1.5 kb fragment containing the gene encoding kanamycin (*kan*) resistance, which is flanked at its 5′ and 3′ ends by a FRT sequence for enabling a FLP catalyzed deletion of the *kan* resistance gene, was isolated from the pKD4 plasmid (Table 5) [106] by PCR using a primer pair *evgSA*_F_ (forward deletion primer) *evgSA*_R_ (reverse deletion primer) with their nucleotide sequences listed in Table 6. The underlined nucleotides in these primers are complementary to nucleotides at 5′ and 3′ ends, respectively, of *evgS* and *evgA* and nucleotides not underlined are complementary to 5′ and 3′ ends, respectively, of the *kan* FRT fragment (Table 6). The procedures for PCR amplification, purification of the amplified DNA fragments, electroporation of the purified DNA fragments in to arabinose-induced competent bacterial cells (strain NADC 6564 in the current study) containing the pKD46 plasmid, selection of *kan*-resistant isolates, removal of the *kan* gene, and confirmation of *evgS/evgA* gene deletion have been described previously [93]. The deletion of *evgS*/*evgA* genes was confirmed by PCR amplification of genomic DNA of kanamycin-sensitive isolates using an *evgSA*_F_ (*evgSA* operon isolation forward primer) and *evgSA*_R_ (*evgSA* operon isolation reverse primer) primer pair as listed in Table 6. These primers were complementary to a short nucleotide sequence located upstream of *evgS* and a short nucleotide sequence located downstream of *evgA*, respectively. The PCR amplified DNA was analyzed by a standard agarose gel electrophoresis to determine the size of the amplified fragments [93].

**Table 6:** Primers used for PCR

Primer	Nucleotide sequence^**a,d**^	Location^**b**^
*gadB*_F_^c^	GTTATCTGGCGTGATGAAGAAG	2688662 - 2688683
*gadB*_R_^c^	GCGTCTAGACATCGACTGCCGTTTGCAGTG	2688757 - 2688738
*gadE*_F_^c^	TGGAGAAATTAGATGCCGAGAG	269494 - 269473
*gadE*_R_^c^	TGATACTTTCTTTGCGGCTAAC	269390 - 269411
*gadX*_F_^c^	CTCAAGGAGGAGGCATTAAATC	262426 - 262443
*gadX*_R_^c^	TTCTTATTCTGCGATAGTTGCG	262544 – 262523
*hdeA*_F_^c^	GTTATTCTTGGTGGTCTGCTTC	271319 - 271340
*hdeA*_R_^c^	GAAATCTTCACAGGTCCAGGAG	271426 - 271405
*evgSA*_F_ (deletion primer)	GAGAAGGGAGATGCTTCATTGCAAAGGGAATAATCTATGAACGGAATCAGATATCTAGCTGACTAAG	1459667 - 1459625
*evgSA*_R_ (deletion primer)	ATAGCTCCCACATTTGAACATTGTGGGAGCCACTATTTAGTTATGAATCAGATATCCTCATCTAGTTAC	1455383 - 1455426
*evgSA*_F_ (*evgSA* operon isolation primer)^e^	CAGAATACATGAAGTTGGTGTG	1455141 - 1455162
*evgS*A_R_ (*evgSA* operon isolation primer)^e^	CCTGTAGGATTAGTGAGAAGAC	1459865 - 1459844

^a^ Nucleotide sequences of primers used in this study were selected from the published genome of *E. coli* O157:H7 strain 6564 [43] with the accession number CP017251.1

^b^ Location refers to the position of primer sequence in the genome of strain 6564

^c^ Subscripts F and R denote forward and reverse primers, respectively

^d^ The underlined represents a portion of the primer sequence corresponding to the indicated location in strain 6564

^e^ These two primers were used for the isolation of the operon containing *evgS* and *evgA* genes and also used in PCR for confirming the deletion of *evgSA* operon in strain 6564

The plasmid for complementing an *evgS*/*evgA* deletion mutation (as constructed above) in strain NADC 6564 was generated by cloning a 4.17 kb DNA fragment containing the *evgS*/*evgA* operon at the *Sma*I site located in the kanamycin gene of a low copy vector pACYC177 (Table 5; New England Biolabs Inc., Ipswich, MA). The 4.17 kb DNA fragment was isolated by PCR amplification of DNA purified from strain NADC 6564 using primers *evgSA*_F_ (*evgSA* operon isolation forward primer) and *evgSA*_R_ (*evgSA* operon isolation reverse primer) as listed in Table 6. Procedures for PCR DNA amplification, purification of amplified DNA fragments, ligating the *Sma*I-linearized 4.17 kb fragment in *Sma*I-linearized pACYC177, transformation of ligated DNA fragments into *E. coli* TOP 10 electrocompetent cells, and confirming the presence of a cloned 4.17 kb fragment in pACYC177 have been described previously [93].

### Bacterial growth curves

The overnight bacterial cultures grown in LB-broth at 37 °C with shaking (200 rpm) were diluted 1:100 in DMEM containing 100 μg per ml of streptomycin. Aliquots (300 μl) of diluted cultures were added to wells of a 100-well Honeycomb 2 plate (Growth Curves USA, Piscataway, NJ). The plate was incubated at 37 °C in an automated growth curve reader for recording optical density at 600 nm (Growth Curves USA, Piscataway, NJ). The growth curve data was collected by analyzing three independently grown bacterial cultures and each culture being assayed in triplicate wells.

### Acid resistance assays and detection of biofilm formation

For determining relative survival of bacterial strains at pH 2.5, three independently grown overnight cultures of each bacterial strain were diluted at 1:1000 in a phosphate-citrate minimal medium (pH 2.5) containing 0.4% glucose and 1.5% sodium glutamate [107, 108]. After 3 h of incubation at 37 °C, the viable bacterial cell counts were determined by plating 10-fold serial dilutions on LB agar medium containing carbenicillin (100 μg per ml). Bacterial survival was calculated by dividing the viable counts at 3 h with the viable counts of the same strain at 0 min. Bacterial survival was plotted as a percent survival.

### Quantitative RT-qPCR

Total DNA-free RNA was prepared from three biological replicates of control and NE-treated bacterial strain NADC 6564 as described above in the section ‘Transcriptional Profiling’**.** The expression of acid resistance pathway 2 (ARP2) encoding genes was determined by transcribing DNA-free RNA into cDNA and amplifying the cDNA using the iTaq Universal One-Step RT-qPCR Kit in CFX96 PCR system according to the manufacturer’s instructions (Bio-Rad, Hercules, CA). The fold change in gene expression was determined using the software and according to the instructions of the manufacturer (Bio-Rad, Hercules, CA). The expression data were normalized to endogenous levels of *rpoA* in order to account for any minor variations in the amounts of RNA across samples [93]. Biofilm formation was detected by crystal violet staining [93, 94] of any potential biofilm produced by strain NADC 6564 grown for 72 h in a biofilm formation-supporting medium containing or lacking NE [93, 94].

### Statistical analyses

Student’s t-test was used to determine the significance of differences in the acid resistance of *evgS/evgA* mutant or *evgS/evgA* mutant complemented with the *evgS/evgA* recombinant plasmid to the parental strain 6564. The difference in growth rate of strain NADC 6564 in the presence or absence of norepinephrine was evaluated by the t-test as described above. Data were analyzed with GraphPad Prism8 (GraphPad Software, La Jolla, CA). The difference was considered significant at *p* < 0.05.

## Supplementary Information

**Additional file 1: Table S1.** List of 5509 genes with reads mapped to the reference genome in response to growth of *E. coli* O157:H7 strain NADC 6564 in response to norepinephrine.

**Additional file 2: Table S2.** List of genes upregulated in response to growth of *E. coli* O157:H7 strain NADC 6564 in response to norepinephrine.

**Additional file 3: Table S3.** List of genes downregulated in response to growth of *E. coli* O157:H7 strain NADC 6564 in the presence of norepinephrine.

**Additional file 4: Fig. S1.** Comparison of the growth rate and viable bacterial cell counts of *E. coli* O157:H7 strain NADC 6564 grown in the absence or presence of norepinephrine. (A) Bacterial growth was measured by taking A_600_ readings over a 24 h period for strain NADC 6564 grown in DMEM lacking (green curve) or containing norepinephrine (red curve). Each growth curve was generated by plotting means (± SD) of A_600_ readings of three independent cultures whereby triplicate of each culture were analyzed for growth and (B) Viable cell counts were determined by plating 10-fold serial dilutions of strain NADC 6564 grown in the absence (green bar) or presence (red bar) of norepinephrine as described in materials and methods. The error bars represent standard deviation of the mean of three independent assays. *** *p* = 0.0005.

## Data Availability

The complete chromosomal sequence of NADC 6564 is available at the GenBank under the assigned accession number CP017251. *E. coli* O157:H7 strain NADC 6564 will be provided pending that the requestor would fulfill requirements for shipment of RG2 bacterial agents. The RNA-Seq raw data is available in the NCBI SRA database under Study SRP091887 comprising accessions SRR16601911 - SRR16601916, which is linked to BioProject PRJNA341860 and BioSamples SAMN22608725 and SAMN22608726.

## Abbreviations

NE: Norepinephrine
RNA: Ribonucleic acid
rRNA: Ribosomal ribonucleic acid
DE: Differentially expressed genes
Fe^3+^: Ferric ion
STEC: Shiga toxigenic *Escherichia coli*
HUS: Hemolytic uremic syndrome
T3SS: type III secretion system
Ler: Locus of enterocyte effacement regulator
GIT: Gastrointestinal tract
QS: Quorum sensing
AHL: acyl-homoserine lactones
ARP2: Acid resistance pathway 2
AI-3: Autoinducer-3
ENS: Enteric nervous system
E: Epinephrine
DHMA: dihydroxymandelic acid
*stx*: Shiga toxin encoding genes
NADC: National Animal Disease Center
DMEM: Dulbecco’s minimal Eagles medium
TCSS: Two component signal transduction system
cdGMP: cyclic diguanylate monophosphate
Usp: Universal stress proteins
PTS: phosphotransferase system
ATP: Adenosine triphosphate
LB: Luria Bertani broth
mg: milligram
A_600_: Absorbance at 600 nm
nm: nanometers
*kan*: Kanamycin
μg: microgram
μl: microliter
cDNA: complementary deoxyribonucleic acid
RT-qPCR: Reverse transcriptase-quantitative polymerase chain reaction
Cfu: Colony forming units
