# Supplementary figures and images for: Genomewide transcriptional response of Escherichia coli O157:H7 to norepinephrine
Source: BMC Genomics. 2022 Feb 8;23:107. doi: 10.1186/s12864-021-08167-z (PMC8822769; doi:10.1186/s12864-021-08167-z)

## Slide 1
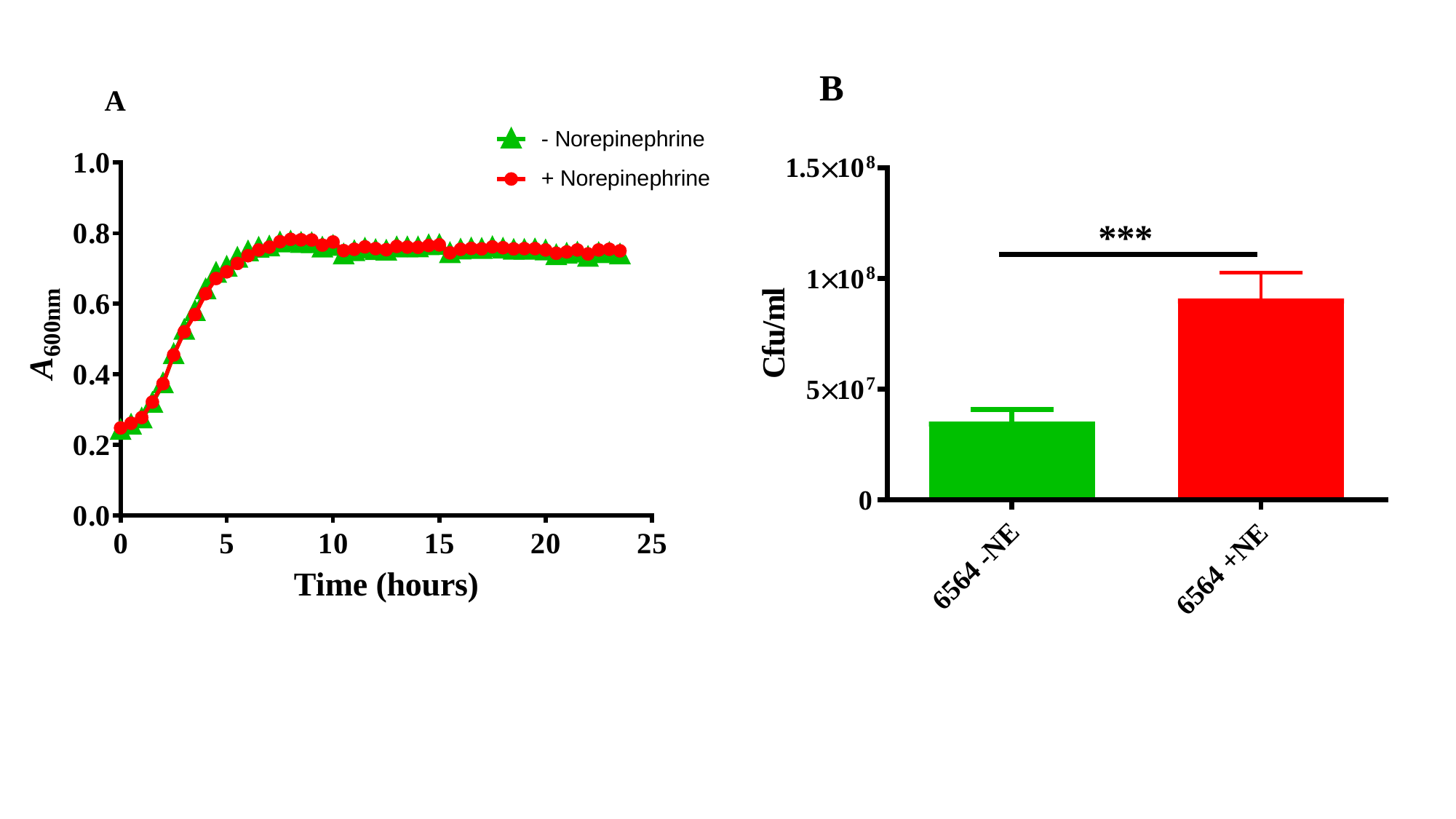

Supplement: Supplementary file 4 — Additional file 4: Fig. S1. Comparison of the growth rate and viable bacterial cell counts of E. coli O157:H7 strain NADC 6564 grown in the absence or presence of norepinephrine. (A) Bacterial growth was measured by taking A600 readings over a 24 h period for strain NADC 6564 grown in DMEM lacking (green curve) or containing norepinephrine (red curve). Each growth curve was generated by plotting means (± SD) of A600 readings of three independent cultures whereby triplicate of each culture were analyzed for growth and (B) Viable cell counts were determined by plating 10-fold serial dilutions of strain NADC 6564 grown in the absence (green bar) or presence (red bar) of norepinephrine as described in materials and methods. The error bars represent standard deviation of the mean of three independent assays. *** p = 0.0005. [file 12864_2021_8167_MOESM4_ESM.pptx]
